# DeepCARS-Identified High-Risk Patients: Clinical Interventions and Outcomes During the Korean Healthcare Crisis

**DOI:** 10.3390/medicina61111896

**Published:** 2025-10-22

**Authors:** Hyojin Jang, Wanho Yoo, Sora Hwang, Kwangha Lee

**Affiliations:** 1 Division of Pulmonary and Critical Care Medicine, Department of Internal Medicine, Pusan National University Hospital, Busan 49241, Republic of Korea; hyoding2@naver.com (H.J.); lcc2202@naver.com (W.Y.); sora0310@nate.com (S.H.); 2 Biomedical Research Institute, Pusan National University Hospital, Busan 49241, Republic of Korea; 3 Department of Internal Medicine, Pusan National University School of Medicine, Busan 49241, Republic of Korea

**Keywords:** artificial intelligence, early warning systems, critical care, resuscitation orders, tertiary care centers

## Abstract

*Background and Objectives*: Timely recognition of deteriorating ward patients is critical to prevent adverse outcomes. The Deep learning–based Cardiac Arrest Risk Score (DeepCARS), an AI-based early warning system developed in Korea, has demonstrated high sensitivity and specificity, but its impact on real-world physician decision-making remains unclear, especially under healthcare resource constraints. *Materials and Methods*: We retrospectively analyzed 830 adult ward patients (March 2024–February 2025) who triggered DeepCARS alerts (score ≥ 91) at a tertiary hospital during a nationwide workforce shortage. Physician responses were classified as active intervention (ICU transfer, life-sustaining treatment [LST] decision, or specialty consultation) versus observation. *Results*: Among patients with DeepCARS ≥ 91, 58.9% received active intervention, with higher in-hospital mortality compared with those observed only (34.8% vs. 9.7%). ROC analysis suggested a cutoff of ≥94 for better intervention discrimination (AUC = 0.708). In multivariable analysis, DeepCARS ≥ 94 (OR 3.52) and chronic liver disease (OR 1.78) independently predicted active intervention. Multinomial analysis showed that patients admitted to medical departments were more often directed toward LST decisions rather than ICU transfer. Hemato-oncologic comorbidities were associated with both ICU transfer and LST decisions, while elevated respiratory rate consistently predicted either ICU transfer or LST discussions. *Conclusions*: DeepCARS alerts effectively triggered physician-driven decisions regarding ICU transfer and end-of-life care during a healthcare crisis. However, the ultimate clinical responses were shaped by comprehensive clinical judgment that integrated AI-generated risks with patient-specific factors, such as functional status and frailty, not captured by the algorithm. This underscores the indispensable role of individualized clinical assessment in interpreting and acting upon AI-based alerts in high-risk ward patients.

## 1. Introduction

Timely recognition and intervention in clinically deteriorating ward patients are essential for preventing adverse outcomes such as unexpected cardiac arrest and unplanned intensive care unit (ICU) admissions. Traditional early warning systems (EWS), including the Modified Early Warning Score (MEWS) and National Early Warning Score (NEWS), rely on simple physiological parameters and have demonstrated utility in detecting at-risk patients. However, these approaches often suffer from limited predictive accuracy and inconsistent responsiveness in real-world practice [1,2,3,4].

To address these limitations, a variety of artificial intelligence (AI)-based EWS have been developed globally, integrating large volumes of electronic medical record (EMR) data to improve predictive performance [5,6,7,8]. The Deep learning–based Cardiac Arrest Risk Score (DeepCARS), developed in Korea, is one such system that has demonstrated high sensitivity and specificity in prior validation studies [9,10,11]. However, high predictive accuracy alone does not guarantee successful clinical implementation, as challenges such as alert fatigue and workflow integration issue remain significant barriers in real-world practice [12]. Therefore, understanding how clinicians actually interpret and respond to these alerts is a crucial, yet understudied, aspect of their clinical utility. Although primarily designed for cardiac arrest prediction, its threshold-based alerts may also influence broader physician decision-making processes, including ICU transfer, specialty consultations, or even limitation-of-care decisions such as life-sustaining treatment (LST) discussions

The predefined threshold of DeepCARS ≥ 91 has been shown in prior work to balance sensitivity and specificity for predicting in-hospital cardiac arrest. In our institution, this cutoff is also embedded in clinical workflow as an actionable alert [9,10,11], providing a pragmatic criterion for identifying patients at heightened risk. However, how clinicians interpret and respond to such alerts in real-world settings—beyond mortality prediction—remains unclear. However, high predictive accuracy in a model does not automatically translate to improved clinical outcomes. The real-world utility of such a system critically depends on how front-line clinicians interpret and act upon its alerts within their complex daily workflow. This raises crucial questions: Do physicians trust the AI-generated recommendations? How are these alerts prioritized amidst numerous other clinical tasks? Answering these questions is essential for understanding the true clinical impact of AI tools beyond their algorithmic performance. This study was conducted during the nationwide healthcare workforce crisis, which provided a unique context to evaluate real-world physician responses under resource constraints, although the development of DeepCARS itself preceded this crisis.

The present study aimed to evaluate physicians’ responses to DeepCARS alerts (≥91) in general ward patients at a tertiary hospital in Korea, during a nationwide healthcare workforce crisis when medical staffing was markedly reduced. Specifically, we examined factors associated with distinct clinical responses, including consultation, ICU transfer, or LST decision-making. By doing so, this study provides insight into the broader clinical utility of AI-based EWS and highlights how patient characteristics, comorbidities, and physiological derangements interact with AI alerts to guide individualized care decisions in practice.

## 2. Materials and Methods

### 2.1. Study Design and Patient Selection

This retrospective observational study was conducted at a single tertiary-care university hospital in South Korea. Prior to the healthcare workforce crisis in February 2024, the hospital operated with 1178 inpatient beds. During the 2024–2025 Korean Medical Crisis, however, the number of functional beds decreased to 880 due to the mass resignation of resident physicians and prolonged leave by medical students.

The DeepCARS system, an AI-driven early warning tool designed to detect in-hospital patient deterioration and predict cardiac arrest, was introduced at this hospital in November 2023 [9,10]. According to this system, when a patient’s DeepCARS score exceeds 91, categorized as high-risk, an automated alert is sent simultaneously via text message to both the responsible attending physician and the nurse station of the relevant ward. Upon receiving the alert, the attending physician is expected to assess the patient and initiate appropriate clinical interventions as needed.

Adult patients admitted from 1 March 2024 to 28 February 2025 were included for analysis. Patients were included if they had been admitted to general wards during the study period, had a DeepCARS score ≥ 91 and had no prior LST decisions. Patients were excluded if they had already been admitted to the ICU at the time of scoring; had prior LST decisions despite a DeepCARS score ≥ 91; or had been hospitalized repeatedly, with LST decisions determined during a previous admission. In such cases, subsequent admissions were excluded from analysis.

The primary outcome of the study was in-hospital mortality, defined as death occurring during the same hospital admission in which the high DeepCARS score was recorded

### 2.2. Ethics Statement

The study protocol was approved by the Institutional Review Board (IRB) of our institute—Pusan National University Hospital Institutional Review Board, which waived the requirement for patient informed consent because of the retrospective and observational nature of the study (IRB No. 2504-011-150, 25 April 2025). All study procedures conformed to the Declaration of Helsinki 2024 and the Strengthening the Reporting of Observational Studies in Epidemiology guidelines [13,14].

### 2.3. Data Collection

Demographic and clinical data, including age, sex, duration of hospital stay, and in-hospital mortality, were collected retrospectively from patients’ electronic medical records. Departments of admission were classified as either medical or surgical. Patients admitted to the internal medicine, neurology, rehabilitation medicine, and emergency medicine departments were categorized as medical admissions, whereas those admitted to the general surgery, orthopedic surgery, neurosurgery, thoracic surgery, trauma surgery, urology, obstetrics and gynecology, ophthalmology, and otorhinolaryngology departments were categorized as surgical admissions. Underlying comorbidities before hospital admission were also recorded, including cardiovascular, hemato-oncologic, chronic lung, chronic kidney, and chronic liver diseases.

For each patient, vital signs and the DeepCARS score were recorded at the first instance the score reached ≥91 at any point during their general ward admission, not necessarily at the time of initial hospital admission. Physician responses were also determined, including consultation with a specialist or multidisciplinary team, transfer to the ICU, LST decisions, and observational management. A ‘life-sustaining treatment (LST) decision’ in this study refers to a formal, legally binding determination made in accordance with the South Korean “Act on Hospice, Palliative Care, and Decisions on Life-Sustaining Treatment for Patients in the Dying Process.” Following consultation between the medical team and the patient (or their guardian), this decision signifies that the patient will not be admitted to the ICU and will forgo all life-sustaining treatments typically provided therein, including cardiopulmonary resuscitation, mechanical ventilation, and renal replacement therapy. The term ‘LST decision’ was retained throughout the manuscript to align with the official terminology of this national legal framework. Patients were categorized into two groups, an active-intervention group or an observation-only group. Patients receiving active physician-directed responses to high DeepCARS scores, including multidisciplinary consultations, transfer to the ICU, or LST decisions, were assigned to the active-intervention group, whereas the remaining patients, who were managed with observation alone, were assigned to the observation-only group. Although ICU transfer, LST decision, and specialty consultation differ in clinical implications, they were grouped as ‘active intervention’ in this study because each represents a physician-driven response beyond observation.

### 2.4. Statistical Analysis

Continuous variables were expressed as mean ± standard deviation or median with interquartile range, and categorical variables as counts and percentages. Continuous variables in two groups were compared using independent *t*-tests or Mann–Whitney U tests, whereas continuous variables in three or more groups were compared using Kruskal–Wallis tests. Categorical variables were compared using the chi-square test or Fisher’s exact test.

Factors predicting active intervention based on DeepCARS scores were evaluated by receiver operating characteristic (ROC) curve analysis, with the optimal cutoff value determined using the Youden index [15].

Predictors of active intervention were examined using multivariable logistic regression. Candidate variables with *p* < 0.05 in univariate comparisons were entered into the model. The Hosmer–Lemeshow test was used to evaluate calibration, and the findings were expressed as odds ratios (ORs) with corresponding 95% confidence intervals (CIs). Statistical significance was defined as *p* < 0.05.

In addition, a multinomial logistic regression analysis was performed to explore predictors distinguishing between the three clinical decisions following high DeepCARS alerts (ICU transfer, LST decision, and consultation only). “LST decision” was used as the reference category for the comparison between ICU transfer and LST, while “consultation only” served as the reference for ICU transfer vs. consultation and LST decision vs. consultation. Independent factors included sex, admission department, and comorbidities, while age, mean blood pressure, pulse rate, respiratory rate, and body temperature were entered as covariates. Model fit was assessed using the −2 log likelihood, likelihood ratio tests, and Nagelkerke R^2^. Results are reported as ORs with 95% CIs. Missing data were handled by complete-case analysis without imputation.

Statistical analyses were performed using IBM SPSS Statistics (version 28.0; IBM Corp., Armonk, NY, USA) and MedCalc version 23.1.6 (MedCalc Software, Ostend, Belgium).

## 3. Results

### 3.1. Patient Characteristics

**Figure 1:**
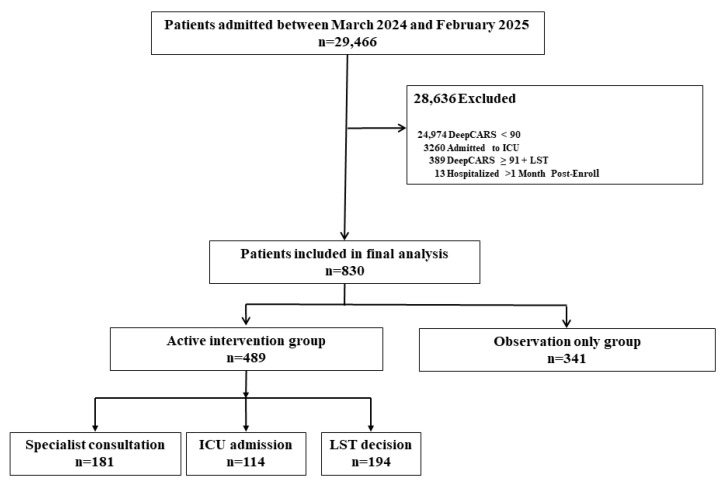
Patient flow chart and classification of patients with DeepCARS scores ≥ 91 into active-intervention and observation-only Groups.

Of the 29,466 patients admitted between March 2024 and February 2025, 830 had DeepCARS scores ≥ 91 and met the inclusion and exclusion criteria (Figure 1). Their mean age was 69.8 years, 61.4% were male, and 70.2% were admitted to medical departments. Hemato-oncologic comorbidities were significantly more frequent in non-survivors than in survivors, whereas neurologic diseases were more common among survivors. The mean DeepCARS score was significantly higher in non-survivors than in survivors (Table 1).

### 3.2. Comparisons of Active Intervention and Observation Alone

Of the 830 included patients, 489 received active intervention and 341 were managed with observation alone (Figure 1). DeepCARS scores, respiratory rate, and pulse rate were significantly higher in the active-intervention group. Hemato-oncologic comorbidities were also more frequent in this group, as was the in-hospital mortality rate (Table 2). 

Most notably, the in-hospital mortality rate in the active-intervention group was strikingly higher than that in the observation-only group (38.2% vs. 1.8%, *p* < 0.001). This strongly suggests that even within the high-risk cohort flagged by a DeepCARS score ≥ 91, clinicians were effectively identifying and intervening in a subgroup of patients who were at an exceptionally high risk of death.

**Figure 2:**
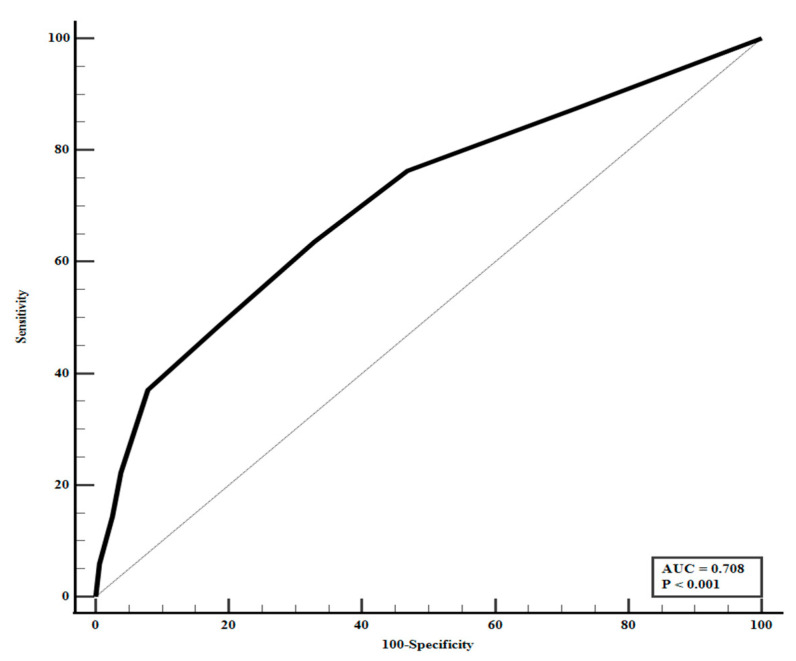
Receiver operating characteristic curve for DeepCARS score in predicting active intervention. The optimal cutoff value of the DeepCARS score for predicting active intervention was 94, with a sensitivity of 63.6% and a specificity of 67.2%. The area under the receiver operating characteristic (AUC) curve was 0.708 (95% CI, 0.676–0.739; *p* < 0.001).

The clinical utility of the DeepCARS score as a trigger for physician intervention was evaluated by ROC curve analysis. A score of 94 was identified as the optimal threshold, demonstrating moderate discriminatory power (area under the curve [AUC] = 0.708; 95% CI, 0.676–0.739; *p* < 0.001), with a sensitivity of 63.6% and a specificity of 67.2% (Figure 2).

### 3.3. Clinical Responses in Subgroups of the Active-Intervention Group

Patients in the active intervention group were classified into three subgroups: those who received consultation alone, those who underwent ICU transfer, and those receiving LST decisions (Table 3). The mortality rate was highest in the LST subgroup (65.5%), followed by the ICU transfer (47.4%) and consultation-only (3.3%) subgroups (*p* < 0.001). Respiratory rate was highest in the ICU transfer group (26 [22–30] breaths/min, *p* < 0.05). Patients in the LST group were more frequently from medical departments (*p* < 0.05).

### 3.4. Predictors of Active Intervention in Total Patients

In the univariate analysis, higher DeepCARS scores (≥94), chronic liver diseases, and hemato-oncologic diseases were significantly associated with receiving intervention. Multivariate logistic regression confirmed that DeepCARS score ≥ 94 remained an independent predictor of intervention (OR 3.517, 95% CI 2.623–4.716, *p* < 0.001). Chronic liver disease (OR 1.782, 95% CI 1.061–2.994, *p* = 0.029) was also independently associated with intervention, whereas hemato-oncologic disease showed significance only in the univariate model (Table 4).

### 3.5. Predictors of Clinical Decisions Following DeepCARS Score ≥ 91

In the multinomial logistic regression analysis, distinct predictors were observed for ICU transfer compared with consultation only. Patients with cardiovascular or hemato-oncologic comorbidities had higher odds of ICU transfer, and elevated heart rate and respiratory rate were also significant predictors. Conversely, patients admitted to medical departments were less likely to be transferred to the ICU.

When ICU transfer was compared with LST decisions, medical admissions showed significantly lower odds of ICU transfer, whereas cardiovascular and hemato-oncologic comorbidities exhibited borderline associations. Physiological parameters, including mean blood pressure, heart rate, and respiratory rate, were not significant predictors in this comparison.

In the comparison of LST decisions with consultation only, hemato-oncologic comorbidities were strongly associated with LST decisions, and medical admissions also showed a preference toward this pathway. Furthermore, lower mean blood pressure and higher respiratory rate were independently associated with increased odds of LST decisions over consultation only. (Table 5).

## 4. Discussion

This study evaluated patients admitted to a tertiary university hospital during a nationwide healthcare workforce crisis, during which medical staffing was significantly reduced. Under these constrained conditions, the real-world clinical application of the DeepCARS system, an AI-based early warning tool originally developed to predict in-hospital cardiac arrest, was evaluated. The findings of this study suggest that the DeepCARS system may not only predict cardiac events but also may provide a practical threshold for initiating physician-driven clinical interventions in high-risk ward patients. To our knowledge, this is the first study in Korea to examine the real-world implementation of DeepCARS in general wards during a period of medical system disruption.

Perhaps the most striking finding of this study was that patients selected for active intervention by clinicians had a dramatically higher mortality rate than those managed with observation alone, despite all patients having a high-risk DeepCARS score. This highlights the profound predictive power of comprehensive clinical judgment, which appears to surpass that of the AI alert itself. As the DeepCARS algorithm is based on quantitative data like vital signs, it has an inherent limitation in that it does not account for crucial prognostic factors such as a patient’s overall functional status, ability to walk, frailty, sarcopenia, or dementia. These non-quantitative factors are often the first things clinicians assess. A high DeepCARS score in a patient with good functional status may have been interpreted by clinicians as a reversible issue, warranting only observation. Conversely, a similar score in a frail, sarcopenic, or cognitively impaired patient may have prompted a decision for intervention, often a limitation of care (LST decision), because the clinician judged the patient’s potential for meaningful recovery to be low. Therefore, our findings affirm that AI-based early warning systems are best viewed as valuable support tools that alert clinicians, rather than as replacements for clinical assessment. The ultimate, nuanced decision-making—which weighs the patient’s complete clinical picture—remains firmly in the domain of human expertise. Future iterations of AI warning systems should aim to incorporate these critical functional and qualitative variables to improve their clinical utility.

Although all patients met the predefined high-risk threshold of DeepCARS ≥ 91, the mean score was significantly higher among those who received active intervention compared with those managed with observation alone. This indicates that clinicians should consider the absolute value of the score, and not just whether the alert is triggered, when determining the need for intervention. Notably, although our institution pragmatically used a cutoff of ≥91 as the actionable threshold, ROC analysis in this study suggested that ≥94 may improve discrimination for predicting physician intervention. This discrepancy underscores the need to continuously refine AI-based thresholds in real-world settings and highlights the critical balance between sensitivity and specificity when integrating AI tools into clinical workflows.

Beyond mortality prediction, our analysis indicated that DeepCARS scores ≥ 94 were associated with a higher likelihood of receiving active intervention, suggesting that this threshold may serve as a useful marker to guide clinical decisions. Comorbidities also appeared to influence physicians’ responses. Chronic liver disease showed an independent association with intervention, while hemato-oncologic comorbidities, although not statistically significant in the multivariate model, were more frequently observed among patients receiving active care. Taken together, these findings suggest that both disease-specific vulnerabilities and physiologic derangements reflected in the DeepCARS score could be related to physicians’ clinical responses in real-world practice.

Interestingly, the patterns of physician decision-making observed in this study were consistent with both baseline characteristics and regression analyses. Patients admitted to medical departments were more often directed toward LST decisions rather than ICU transfer, reflecting the clinical context in which these patients were typically managed. Beyond admission type, specific comorbidities also appeared to shape the direction of response: those with cardiovascular diseases were more frequently considered for ICU transfer compared with consultation alone, while patients with hemato-oncologic conditions were more likely to proceed either to ICU transfer or to LST decisions. Physiological derangements further contributed to these choices, with elevated respiratory rate emerging as a common signal prompting both ICU transfer and LST discussions. Together, these findings suggest that physician responses following high-risk alerts are not determined by a single factor but reflect an interplay between underlying vulnerabilities, the admitting department, and acute physiological changes.

About 40% of patients in this study with high DeepCARS scores were managed conservatively without active intervention. Notably, these patients showed relatively low mortality, indicating that in some cases alerts did not necessitate escalation. This suggests that certain alerts may reflect transient or reversible abnormalities, and that clinicians appropriately exercised discretion in filtering meaningful signals from noise. These observations emphasize that while AI-based systems can provide valuable guidance, their optimal role is to support, rather than replace, individualized clinical judgment.

Importantly, this study was conducted during a nationwide healthcare workforce crisis, which provided a unique stress test for the DeepCARS system. Physician decision-making under severe resource constraints may differ from usual practice, yet the emergence of consistent and interpretable patterns of response in this setting suggests the robustness of AI-based alerts even under strained healthcare environments. This context strengthens the generalizability of our findings, as it illustrates how AI tools may function as decision-support mechanisms during both routine practice and periods of systemic disruption.

These findings underscore the importance of viewing AI-generated alerts as part of a broader decision-making process, rather than as stand-alone triggers. Building on this perspective, further research is needed to define optimal thresholds for AI-triggered responses, clarify the role of clinical judgment in these responses, and evaluate the applicability of early warning systems such as DeepCARS in different hospital environments and patient populations. Prospective multicenter studies that incorporate response time, patient outcomes, and clinician perspectives will be essential to establish standardized pathways for effectively integrating AI alerts into routine clinical workflows [12]. 

This study has several limitations. First, it was conducted at a single tertiary-care hospital during a nationwide healthcare workforce crisis, which may limit the generalizability of the findings to other settings or to periods of normal staffing. The reduced availability of physicians during this period may have influenced both the timeliness and the nature of responses to DeepCARS alerts. Second, the retrospective design introduces inherent limitations, including the potential for selection bias and unmeasured confounding. Third, clinical responses following DeepCARS alerts—such as intervention, ICU transfer, or LST decisions—were not standardized and may have varied depending on physician judgment and clinical circumstances. Finally, due to the retrospective design of this study, we were unable to collect precise data on the time interval between the DeepCARS alert and subsequent clinical outcomes, such as ICU transfer or in-hospital death. This is a notable limitation, as this temporal data could have provided further insight into whether the timing of a patient’s deterioration (e.g., early vs. late in the hospital stay) influenced the type of clinical response, such as the decision between ICU transfer and an LST decision. 

## 5. Conclusions

In conclusion, this study demonstrated the effectiveness of the DeepCARS system as a clinical decision-support tool during a healthcare crisis. Its primary value was serving as a reliable trigger that prompted physicians to make critical and timely clinical decisions—ranging from ICU transfer to end-of-life care planning—for appropriately identified high-risk patients.

## Figures and Tables

**Table 1:** Clinical characteristics of all enrolled patients and comparisons in survivors and non-survivors.

Characteristic	Total (n = 830)	Hospital Mortality		*p* Value
		Survivors (n = 637)	Non-Survivors (n = 193)	
Demographics				
Age, years, mean ± SD	69.8 ± 12.2	69.6 ± 12.2	70.4 ± 12.7	0.442
Male sex, n (%)	510 (61.4)	390 (61.2)	120 (62.2)	0.866
Admission department, n (%)				
Medical	583 (70.2)	448 (70.3)	135 (69.9)	0.929
Surgical	247 (29.8)	189 (29.7)	58 (30.1)	
Hospital length of stay, days, median (IQR)	16 (8–29)	15 (8–29)	18 (7–30)	0.507
Underlying comorbidities before admission, n (%)				
Cardiovascular diseases	534 (64.3)	411 (64.5)	123 (63.7)	0.864
Hemato-oncologic diseases	513 (61.8)	368 (57.8)	145 (75.1)	<0.001
Diabetes mellitus	292 (35.2)	231 (36.3)	61 (31.6)	0.263
Chronic lung diseases	140 (16.9)	110 (17.3)	30 (15.5)	0.661
Chronic kidney diseases	113 (13.6)	89 (14.0)	24 (12.4)	0.633
Chronic liver diseases	83 (10.0)	59 (9.3)	24 (12.4)	0.218
Neurologic diseases	21 (2.5)	20 (3.1)	1 (0.5)	0.038
Immunosuppressive state	18 (2.2)	16 (2.5)	2 (1.0)	0.272
Rheumatologic	17 (2.0)	15 (2.4)	2 (1.0)	0.386
DeepCARS score, mean ± SD	93.9 ± 2.3	93.5 ± 2.1	95.2 ± 2.5	<0.001
DeepCARS score ≥ 91 during after-hours duty, n (%)	567 (68.3)	436 (68.4)	131 (67.9)	0.930

Continuous variables presented as mean ± standard deviation (SD) or median (interquartile range [IQR]) and categorical variables as number (percent).

**Table 2:** Comparison of clinical characteristics and vital signs at the time of DeepCARS score ≥ 91 in the active-intervention and observation-only groups.

Characteristic	Intervention Group (n = 489)	Observation Only Group (n = 341)	*p* Value
Demographics			
Age, years, mean ± SD	70.3 ± 12.3	69.1 ± 12.1	0.163
Male sex, n (%)	313 (64.0)	197 (57.8)	0.071
Admission department, n (%)			
Medical	336 (68.7)	247 (72.4)	0.280
Surgical	153 (31.3)	94 (27.6)	
DeepCARS score ≥ 91 during after-hours duty, n (%)	327 (66.9)	240 (70.4)	0.290
Hospital length of stay, days, median (IQR)	18 (9–32)	13 (7–24)	<0.001
Underlying comorbidities before admission, n (%)			
Cardiovascular diseases	312 (63.8)	222 (65.1)	0.713
Hemato-oncologic diseases	316 (64.6)	197 (57.8)	0.046
Diabetes mellitus	164 (33.5)	128 (37.5)	0.238
Chronic lung diseases	80 (16.4)	60 (17.6)	0.639
Chronic kidney diseases	62 (12.7)	51 (15.0)	0.356
Chronic liver diseases	59 (12.1)	24 (7.0)	0.019
Neurologic diseases	11 (2.2)	10 (2.9)	0.654
Immunosuppressive state	10 (2.0)	8 (1.0)	0.811
Rheumatologic	10 (2.0)	7 (2.1)	>0.999
DeepCARS score, mean ± SD	94.6 ± 2.4	92.9 ± 1.8	<0.001
Vital signs at DeepCARS score ≥ 91, median (IQR) ^(a)^			
Systolic blood pressure, mmHg (n = 464/n = 310)	107 (90–127)	100 (90–120)	0.183
Diastolic blood pressure, mmHg (n = 464/n = 309)	61 (54–80)	60 (52–72)	0.338
Pulse rate, /min (n = 461/n = 307)	114 (101–128)	110 (98–121)	<0.001
Respiratory rate, /min (n = 454/n = 280)	24 (20–28)	20 (20–22)	<0.001
Body temperature (axilla), °C (n = 451/n = 287)	36.5 (36.3–37.2)	36.5 (36.3–37.1)	0.725
SpO_2_, % (n = 453/n = 272)	96 (95–98)	97 (95–98)	0.929
In-hospital mortality, n (%)	187 (38.2)	6 (1.8)	<0.001

Continuous variables presented as mean ± standard deviation (SD) or median (interquartile range [IQR]) and categorical variables as number (percent). (a) n values for each vital sign (Active intervention/Observation only) differ because some data were missing at the time of DeepCARS score ≥ 91.

**Table 3:** Comparison of patient characteristics following DeepCARS score ≥ 91 in the three subgroups of the intervention group.

Characteristic	Consultation Only (n = 181)	ICU Transfer (n = 114)	Life-Sustaining Treatment Decision (n = 194)
Demographics			
Age, years, mean ± SD	69.9 ± 12.8	68.6 ± 13.9	71.5 ± 10.8 *
Male sex, n (%)	113 (62.4)	79 (69.3)	121 (62.4)
Admission department: medical, n (%)	118 (65.2)	60 (52.6)	158 (81.4) *
Hospital length of stay, median (IQR)	17 (10–35)	24 (14–35) *	14 (7–28)
Underlying comorbidities before admission, n (%)			
Cardiovascular diseases	116 (64.1)	81 (71.1)	115 (59.3)
Hemato-oncologic diseases	99 (54.7)	66 (57.9)	151 (77.8) *
Diabetes mellitus	65 (35.9)	35 (30.7)	64 (33.0)
Chronic lung diseases	24 (13.3)	20 (17.5)	36 (18.6)
Chronic kidney diseases	19 (10.6)	21 (18.4)	22 (11.3)
Chronic liver diseases	23 (12.7)	16 (14.0)	20 (10.3)
Neurologic diseases	3 (1.7)	4 (3.5)	4 (2.1)
Rheumatologic	3 (1.7)	4 (3.5)	3 (1.5)
Immunosuppressive state	7 (3.9)	2 (0.4)	1 (0.2)
DeepCARS score, mean ± SD	94.0 ± 2.2	95.1 ± 2.5 *	94.8 ± 1.4 *
Vital signs at DeepCARS score ≥ 91, median (IQR) ^(a)^			
Systolic blood pressure, mmHg (n = 464/n = 310)	110 (94–122)	100 (90–130)	109 (90–127)
Diastolic blood pressure, mmHg (n = 464/n = 309)	63 (57–80)	61 (53–80)	60 (52–77)
Pulse rate, /min (n = 461/n = 307)	113 (98–127)	116 (102–132)	115 (102–125)
Respiratory rate, /min (n = 454/n = 280)	22 (20–25)	26 (22–30) *	24 (20–28)
Body temperature (axilla), °C (n = 451/n = 287)	36.5 (36.3–37.4)	36.3 (36.3–37.0)	36.5 (36.3–37.1)
SpO_2_, % (n = 453/n = 272)	97 (95–99)	96 (95–99)	96 (95–98)
DeepCARS score ≥ 91 during after-hours duty, n (%)	120 (66.3)	72 (63.2)	135 (69.6)
Hospital mortality, n (%)	6 (3.3)	54 (47.4)	127 (65.5) *

Continuous variables presented as mean ± standard deviation (SD) or median (interquartile range [IQR]) and categorical variables as number (percent). (a) The number of patients (n) may vary across variables because some data were missing at the time of DeepCARS score ≥ 91. * *p* < 0.05 vs. other subgroups. Abbreviations: ICU = intensive care unit.

**Table 4:** Univariate and multivariate of factors associated with active intervention in total enrolled patients.

Variables	Univariate Analysis		Multivariate Analysis	
	OR (95% CI)	*p* Value	OR (95% CI)	*p* Value
DeepCARS Score ≥ 94	3.572 (2.669–4.782)	<0.001	3.517 (2.623–4.716)	<0.001
Chronic liver diseases	1.812 (1.103–2.977)	0.019	1.782 (1.061–2.994)	0.029
Hemato-oncologic diseases	1.335 (1.005–1.773)	0.046		

Variables with *p* < 0.05 in univariate analyses were included in the multivariate binary logistic regression analysis. Model calibration was assessed using the Hosmer–Lemeshow goodness-of-fit test: chi-square = 0.870, df = 4, *p*-value = 0.929; OR = odds ratio; CI = confidence interval.

**Table 5:** Multinomial logistic regression analysis of factors associated with ICU transfer, LST decision and consultation only in patients with DeepCARS Score ≥ 91.

Variables	ICU Transfer vs. Consultation Only (Ref = Consultation Only)		ICU Transfer vs. LST Decision (Ref = LST Decision)		LST Decision vs. Consultation Only (Ref = Consultation Only)	
	OR (95% CI)	*p* Value	OR (95% CI)	*p* Value	OR (95% CI)	*p* Value
Admission department: Medical	0.479 (0.272–0.844)	0.011	0.237 (0.132–0.424)	<0.001	1.996 (1.161–3.433)	0.012
Male sex	1.310 (0.730–2.352)	0.366	1.770 (0.990–3.165)	0.054	0.736 (0.453–1.195)	0.215
Comorbidity: Cardiovascular diseases	2.215 (1.140–4.302)	0.019	1.723 (0.910–3.262)	0.095	1.268 (0.743–2.165)	0.383
Comorbidity: Hemato-oncologic diseases	1.929 (1.061–3.508)	0.031	0.538 (0.289–1.003)	0.051	3.576 (2.071–6.174)	<0.001
Comorbidity: Diabetes mellitus	0.696 (0.383–1.263)	0.233	0.794 (0.439–1.439)	0.447	0.878 (0.530–1.456)	0.614
Comorbidity: Chronic lung diseases	1.065 (0.489–2.318)	0.875	0.813 (0.388–1.704)	0.583	1.254 (0.649–2.422)	0.501
Comorbidity: Chronic kidney diseases	2.200 (0.981–4.936)	0.056	1.780 (0.801–3.955)	0.157	1.240 (0.575–2.673)	0.583
Comorbidity: Chronic liver diseases	1.223 (0.486–3.079)	0.669	1.629 (0.665–3.990)	0.285	0.765 (0.357–1.640)	0.583
Comorbidity: Neurologic diseases	3.003 (0.368–24.522)	0.305	1.275 (0.183–8.866)	0.806	2.508 (0.375–16.773)	0.343
Comorbidity: Immunosuppressive state	0.740 (0.124–4.430)	0.742	3.094 (0.232–41.237)	0.393	0.246 (0.028–2.166)	0.206
Comorbidity: Rheumatologic diseases	3.140 (0.451–21.885)	0.248	1.695 (0.299–9.610)	0.551	1.832 (0.276–12.163)	0.531
Age, year	0.990 (0.967–1.013)	0.393	0.978 (0.954–1.001)	0.065	1.013 (0.992–1.035)	0.235
Mean blood pressure, mmHg	0.984 (0.968–1.000)	0.055	1.000 (0.984–1.016)	0.997	0.984 (0.970–0.988)	0.030
Pulse rate, /min	1.016 (1.002–1.148)	0.022	1.009 (0.996–1.022)	0.178	1.006 (0.994–1.019)	0.313
Respiratory rate, /min	1.098 (1.051–1.148)	<0.001	1.033 (0.997–1.070)	0.075	1.064 (1.021–1.109)	0.003
Body temperature (axilla), °C	1.003 (0.836–1.203)	0.974	0.996 (0.825–1.202)	0.968	1.003 (0.894–1.125)	0.961

Reference categories: Consultation only (for ‘ICU transfer vs. Consultation only’ and ‘LST vs. Consultation only’) and LST (for ‘ICU transfer vs. LST’). Covariates included in the model: age, mean blood pressure, pulse rate, respiratory rate, and body temperature. Model fit: Nagelkerke R^2^ = 0.238; −2 Log likelihood = 829.602; likelihood ratio test indicated the model was statistically significant (*p* < 0.001). Abbreviations: ICU = intensive care unit; LST = life-sustaining treatment; OR = odds ratio; CI = confidence interval. Statistical significance was set at *p* < 0.05.

## Data Availability

The data presented in this study are available upon request from the corresponding author. The data are not publicly available due to privacy regulations.

## Abbreviations

The following abbreviations are used in this manuscript:

DeepCARS	Deep learning–based Cardiac Arrest Risk Score
ICU	Intensive care unit
EWS	early warning systems
MEWS	Modified Early Warning Score
NEWS	National Early Warning Score
AI	artificial intelligence
LST	life-sustaining treatment
IRB	Institutional Review Board
ROC	receiver operating characteristic
OR	odds ratio
CI	confidence interval
AUC	area under the curve
